# Green Synthesis, Characterization and Uses of Palladium/Platinum Nanoparticles

**DOI:** 10.1186/s11671-016-1695-z

**Published:** 2016-11-02

**Authors:** Khwaja Salahuddin Siddiqi, Azamal Husen

**Affiliations:** 1 Department of Chemistry, Aligarh Muslim University, Aligarh, 202002 Uttar Pradesh India; 2 Department of Biology, College of Natural and Computational Sciences, University of Gondar, PO Box #196, Gondar, Ethiopia

**Keywords:** Biogenic fabrication, Herbal extract, Phytochemicals, Metal nanoparticles, Cancer

## Abstract

Biogenic synthesis of palladium (Pd) and platinum (Pt) nanoparticles from plants and microbes has captured the attention of many researchers because it is economical, sustainable and eco-friendly. Plant and their parts are known to have various kinds of primary and secondary metabolites which reduce the metal salts to metal nanoparticles. Shape, size and stability of Pd and Pt nanoparticles are influenced by pH, temperature, incubation time and concentrations of plant extract and that of the metal salt. Pd and Pt nanoparticles are broadly used as catalyst, as drug, drug carrier and in cancer treatment. They have shown size- and shape-dependent specific and selective therapeutic properties. In this review, we have discussed the biogenic fabrication of Pd/Pt nanoparticles, their potential application as catalyst, medicine, biosensor, medical diagnostic and pharmaceuticals.

## Review

### Introduction

The main aim of green synthesis is to minimize the use of toxic chemicals to prevent the environment from pollution. The biogenic routes for the fabrication of nanomaterials are therefore becoming more and more popular.

The three main conditions for nanomaterials preparation are (i) the choice of environment-friendly solvent medium, (ii) reducing agent and (iii) a nontoxic material for their stabilization. Nanomaterials fabricated from plants, fungi and bacteria have several potential applications in all fields of science and technology [1–10]. The reduction of metal ions occur by the proteins, amines, amino acids, phenols, sugars, ketones, aldehydes and carboxylic acids present in the plants and microbes. The geometrical shape, size and stability of nanoparticles may be controlled by monitoring the pH, temperature, incubation time and concentrations of plant extract and that of the metal salt.

**Fig. 1:**
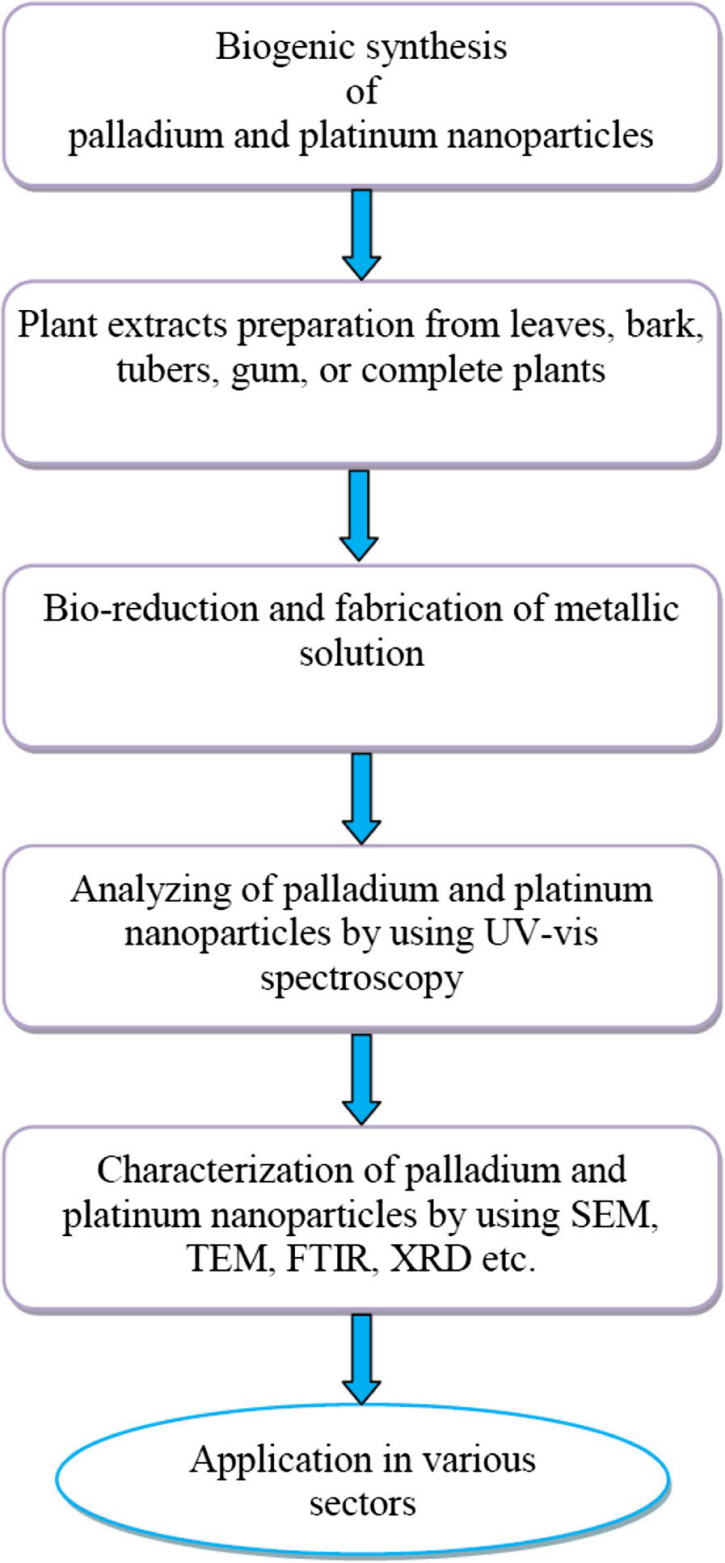
Biogenic synthesis of palladium and platinum nanoparticles

**Fig. 2:**
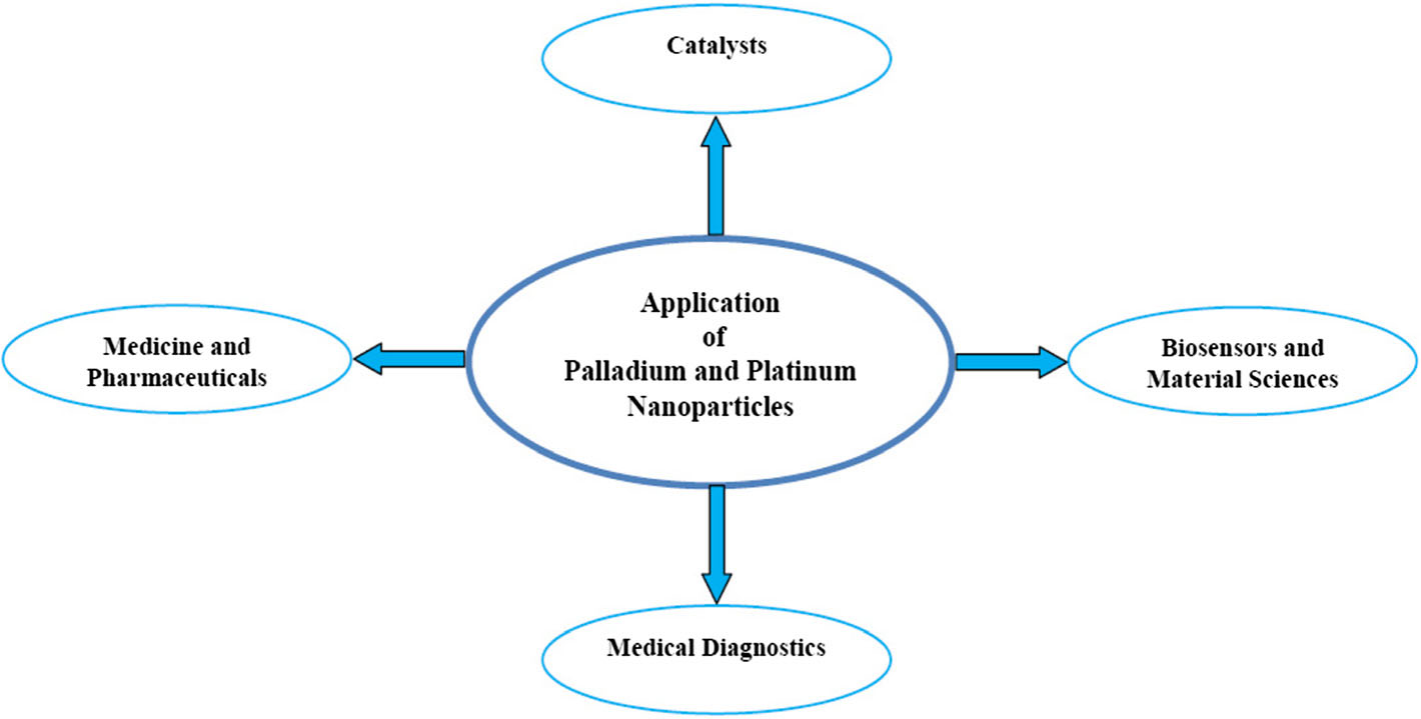
Application of palladium and platinum nanoparticles

Both palladium and platinum are high-density silvery white precious metals. Biogenic fabrication of palladium and platinum nanoparticles using various plant species such as *Anogeissus latifolia*, *Cinnamom zeylanicum*, *Cinnamomum camphora*, *Curcuma longa*, *Doipyros kaki*, *Gardenia jasminoides*, *Glycine max*, *Musa paradisica*, *Ocimun sanctum*, *Pinus resinosa* and *Pulicaria glutinosa* have been reported. The properties of fabricated palladium and platinum nanoparticles using various plants parts are summarized in Table 1 and Figs. 1 and 2. They are employed both as heterogeneous and homogeneous catalysts due to their large surface-to-volume ratio and high surface energy [11]. They are used in many medical diagnoses without destructing the DNA structure [12]. Palladium and platinum nanoparticles fabricated from herbal extracts have been examined for their heterogeneous catalytic activity in Suzuki–Miyaura coupling reaction [13]. Since it is a ligand-free catalytic reaction, it can be easily carried out in an aqueous medium in open without the fear of dissociation. The yield is very high even with one mole % palladium and platinum nanoparticles under ordinary condition.

**Table 1:** Important example of phytosynthesis of palladium and platinum nanoparticles with their size and shape

Plant	Part used	Nanoparticles	Size (nm)	Shape	References
*Anogeissus latifolia*	Gum	Pd	4.8	Spherical	[39]
*Azadirachta indica*	Leaves	Pt	5–50	Small and large spheres	[62]
*Cinnamom zeylanicum*	Bark	Pd	15–20	Crystalline	[23]
*Cinnamomum camphora*	Leaves	Pd	3.2–6.0	–	[86]
*Curcuma longa*	Tuber	Pd	10–15	Spherical	[26]
*Doipyros kaki*	Leaves	Pt	2–12	Crystalline	[26]
*Euphorbia granulate*	Leaves	Pd	25–35	–	[44]
*Gardenia jasminoides*	Leaves	Pd	3–5	–	[27]
*Glycine max*	Leaves	Pd	15	Spherical	[34]
*Moringa oleifera*	Waste petal	Pd	10–50	Spherical	[42]
*Moringa oleifera*	Peel extract	Pd	27 ± 2	Spherical	[43]
*Musa paradisica*	Peeled banana	Pd	50	Crystalline irregular	[33]
*Ocimun sanctum*	Leaves	Pt	23	Irregular	[55]
*Pulicaria glutinosa*	Whole plant	Pd	20–25	Crystalline and spherical	[35]
*Pinus resinosa*	Bark	Pd	16–20	Crystalline	[58]
*Pinus resinosa*	Bark	Pt	6–8	Irregular	[45]
*Prunus* x *yedoensis*	Leaves	Pd	50–150	Spherical	[45]

In this review, we have discussed the biosynthesis of palladium/platinum nanoparticles and their characterization using scanning electron microscopy (SEM), transmission electron microscopy (TEM), X-ray diffraction (XRD), UV-vis and Fourier transform infrared (FTIR) spectroscopy. In addition, their application in catalysis, treatment of cancer and other disciplines of biological sciences has been assessed.

### Biosynthesis of Palladium Nanoparticles

When an aqueous solution of [Pd(OAc)_2_] was stirred with a methanolic extract of *Catharanthus roseus* for 1 h at 60 °C, a change in colour occurred. It showed absorption peak in 360–400 nm range in UV-visible spectrum which corresponds to spherical palladium nanoparticles of ~40 nm. *C. roseus* extract is a mixture of eight compounds containing –OH groups which reduce the metal ion to metal nanoparticles.

$$ \mathrm{P}\mathrm{d}{\left({\mathrm{CH}}_3\mathrm{C}\mathrm{O}\mathrm{O}\right)}_2+\mathrm{Reducing}\kern0.5em \mathrm{extract}\to \mathrm{P}\mathrm{d}+2{\mathrm{CH}}_3\mathrm{CO}\mathrm{O}\mathrm{H} $$

Synthesis, characterization and application of palladium nanoparticles as photocatalytic agent have been reported [14, 15]. The degradation of phenol red by palladium nanoparticles has been investigated. The nanoparticles were added to phenol red and stirred at room temperature at varying pH (2–10). The surface plasmon resonance (SPR) band of dye at 433 nm disappeared at pH 6 showing the degradation of phenol red [15].

Palladium nanoparticles synthesized from aqueous leaf extract of *Hippophae rhamnoides* have been reported [13]. They have been characterized by SEM, TEM, XRD, UV-vis and FTIR spectroscopy. The presence of polyphenols indicated that they act as reducing and capping agents for the palladium nanoparticles. The particle size ranged between 2.5 and 14 nm and most of them were spherical. Their catalytic activity as heterogeneous catalyst was evaluated for Suzuki–Miyaura coupling reaction in water under lignin-free conditions. Iodobenzene with phenylboronic acid in the presence of palladium nanoparticles at 100 °C in alkaline medium gave 100 % yield of the product. Different aryl halides with phenylboronic acid were tried, and all of them gave the corresponding compounds in high yield (91–95 %).

**Fig. 3:**
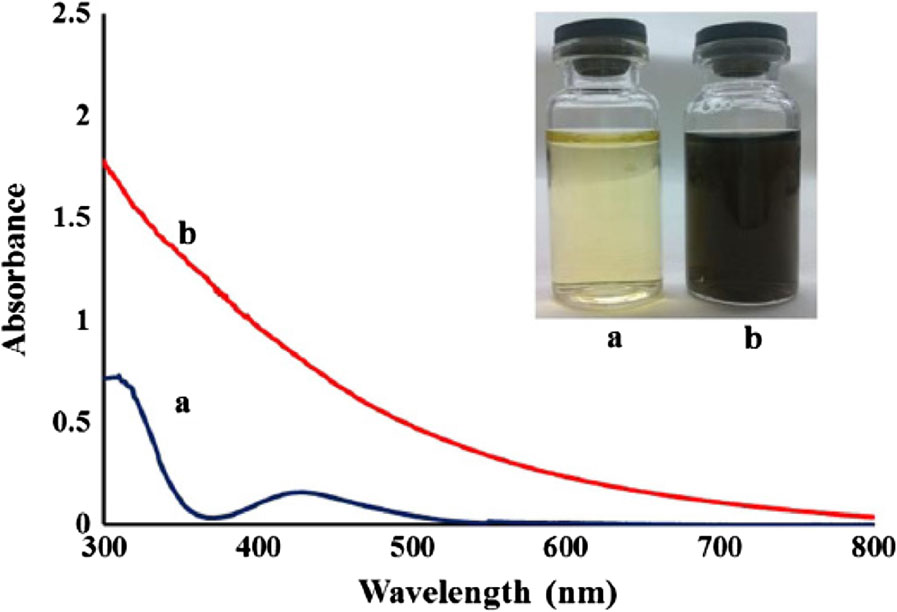
UV-vis spectroscopy of (*a*) PdCl_2_ solution and (*b*) palladium nanoparticles after reduction by crude extract of *Sargassum bovinum* at 60 °C for 24 h. The *inset* shows an image of the as-prepared Pd colloidal solution and the PdCl_2_ solution before reaction [16]

Momeni and Nabipour [16] used *Sargassum bovinum* alga for palladium nanoparticles fabrication. Authors observed the conversion of palladium ions into metallic palladium using UV-vis spectroscopy in the range of 300–800 nm (Fig. 3). Change in colour from yellow to dark brown indicated the formation of palladium nanoparticles. Figure 3 represents the absorption spectra of palladium nanoparticles after 24 h of reduction from the crude extract and compared with those of PdCl_2_ solution. The palladium nanoparticles of 5–10 nm were checked for catalytic activity by electrochemical reduction of H_2_O_2_. Since they were stable up to 5 months, it was believed that they were stabilized by polysaccharides present in the algal extract. The reduction of H_2_O_2_ was also confirmed by cyclic voltametry.

Bimetallic nanoparticle with core-shell structure and shape-controlled synthesis has been reported for Au@Pd nanoparticles [17, 18]. To a reduced gold nanoparticle, another metal was added and subsequently reduced chemically or by plant extract containing a mild reducing agent (*Cacumen platycladi* leaf extract). The gold nanoparticles were enveloped by the second metal nanoparticles giving a particular shape which depends on the arrangement of the second metal nanoparticles around gold. The bimetallic flower-shaped Au@Pd nanoparticles can be seen from a dark central core surrounded by a light colour shell. Their average size ranged between 47.8 ± 2.3 nm with face-centred cubic structure [19].

**Fig. 4:**
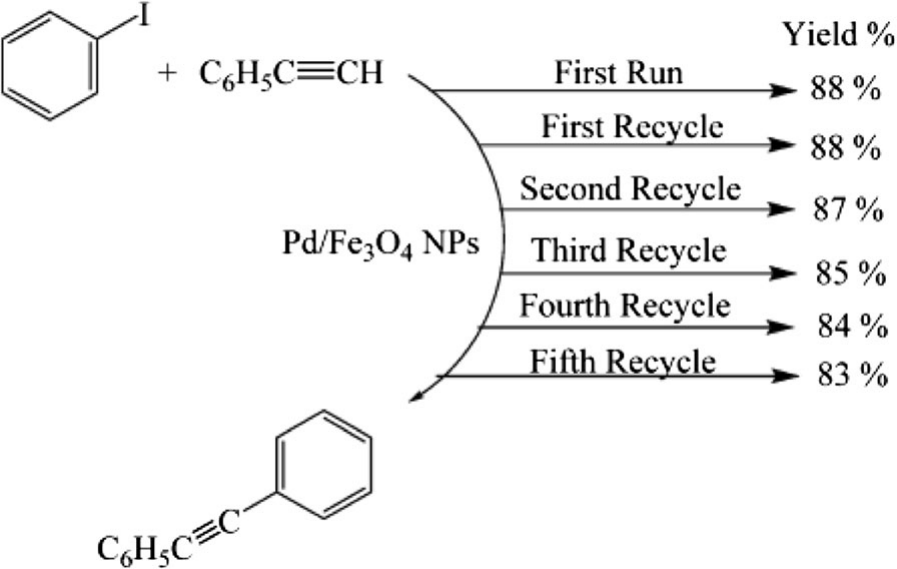
Reusability of Pd/Fe_3_O_4_ nanoparticles for Sonogashira coupling reaction [20]

Green synthesis of Pd/Fe_3_O_4_ nanoparticles from *Euphorbia condylocarpa* M. *bieb* root extract and their catalytic activity have recently been reported [20]. The extract contains flavonoids which provide electrons for the reduction of metal ions. The Fe_3_O_4_/Pd is a good catalyst and can be used for several cycles for Sonogashira and Suzuki coupling reactions without loss of activity, but Fe_3_O_4_ is highly sensitive to air. Since Pd and Fe are magnetic, they were recovered from the reaction mixture by a magnet and recycled several times for Sonogashira coupling reaction with negligible loss of activity (Fig. 4).

Biosynthesis of palladium nanoparticles on reduced graphene oxide using barberry fruit extract and their application as a heterogeneous catalyst for the reduction of nitroarenes to amines has been done at 50 °C in 1:2 alcohol–water mixture [21]. Vitamin C appears to be a major phytochemical in the extract, and therefore, it reduced the metal ions to nanoparticles. The average size of palladium nanoparticles was found to be nearly 18 nm. Catalytic activity was determined by the reduction of nitrobenzene to aniline with NaBH_4_. The reduction occurs on the surface of catalyst and depends on the speed of absorption of nitrocompounds on the active site of the catalyst. The process is complicated but occurs stepwise. Adsorption of H_2_- and nitrocompounds, followed by electron transfer from BH_4_^−^ unit to nitroderivative and finally, desorption of amino compounds from the surface of catalyst occur. The catalyst can be used for five cycles without significant loss of activity.

Very recently, palladium nanoparticles were synthesized from *Salvadora persica* root extract. Extract was found to contain polyphenols which acted both as bioreductant and stabilizing agent [22]. The average nanoparticles of 10 nm at 90 °C were obtained which was ascertained from the loss of colour and disappearance of an absorption band at 415 nm in UV-vis spectrum of the colloidal solution.

Palladium nanoparticles have been synthesized from *C. zeylanicum* bark extract and PdCl_2_ at 30 °C [23]. Although, reaction started after 24 h, it was completed after 72 h. The nanoparticles were polydispersed, spherical in shape ranging between 15 and 20 nm. Their formation was dependent on the increasing concentration of leaf extract. The XRD pattern confirmed the presence of crystalline palladium. The effect of pH on the formation of nanoparticles is insignificant, but precipitation occurs above pH 5. However, it does not influence the shape of nanoparticles but slightly affect their size [24]. It was noticed that nearly 60 % of PdCl_2_ was reduced to palladium nanoparticles when only 5-ml extract was treated with 50 ml of 1 mM PdCl_2_ at 30 °C. Higher concentration of the biomaterial may reduce the remaining 40 % PdCl_2_; otherwise, the suspension would contain both the Pd^2+^ ions and palladium nanoparticles. The *C. zeylanicum* bark extract is known to contain linalool, eugenol, methyl chavicol, cinnamaldehyde, ethyl cinnamate and β-caryophyllene [25] which have distinct aroma and convert Pd ions to Pd nanoparticles. However, no clear mechanism has been given for the reduction process of PdCl_2_ to Pd nanoparticles.

Sathishkumar et al. [26] have reported the biosynthesis of palladium nanoparticles from *C. longa* extract. The nanoparticles of 10–15 nm are believed to be formed by a redox process involving polyphenols as the reducing agent. They were found to be stable even after 3 months. The pH of the solution had almost negligible effect on the formation of nanoparticles, but size increases with pH.

Green synthesis of palladium nanoparticles from dried fruit extract of *G. jasminoides* Ellis has been achieved at 60 °C after 1.5 h of incubation [27]. Formation of nanoparticles was indicated by a change in colour from orange to dark brown. The extract had three distinct absorption peaks at 238, 322 and 440 nm corresponding to geniposide [28], chlorogenic acid [29] and crocins/crocetin [30], respectively. These compounds are antioxidants [30–32] and contain carbonyl, carboxyl and hydroxyl groups. The orange colour was mainly due to crocins/crocetin which disappeared after 1.5 h although the other absorptions did not change even after 12 h at this temperature. The XRD pattern showed the presence of face-centred cubic structure of Pd^0^, with 3.9-nm diameter. The FTIR spectra showed the presence of various functional groups. Some new peaks were detected after the reduction of Pd^2+^ to Pd^0^. Since all Pd^2+^ ions are not completely reduced, the appearance of new peaks was attributed to its coordination with the carbonyl compounds present in the extract. The TEM images showed spherical, rod and three-dimensional polyhedral structures at 40 °C, but they vary with increasing temperature. The smaller particles are nicely dispersed at 70 °C while the larger ones are agglomerated. Normally, the particles size varies between 4.47 and 13.63 nm at temperature between 40 and 90 °C although more than 75 % of the palladium nanoparticles were 3–5-nm diameter.

**Fig. 5:**
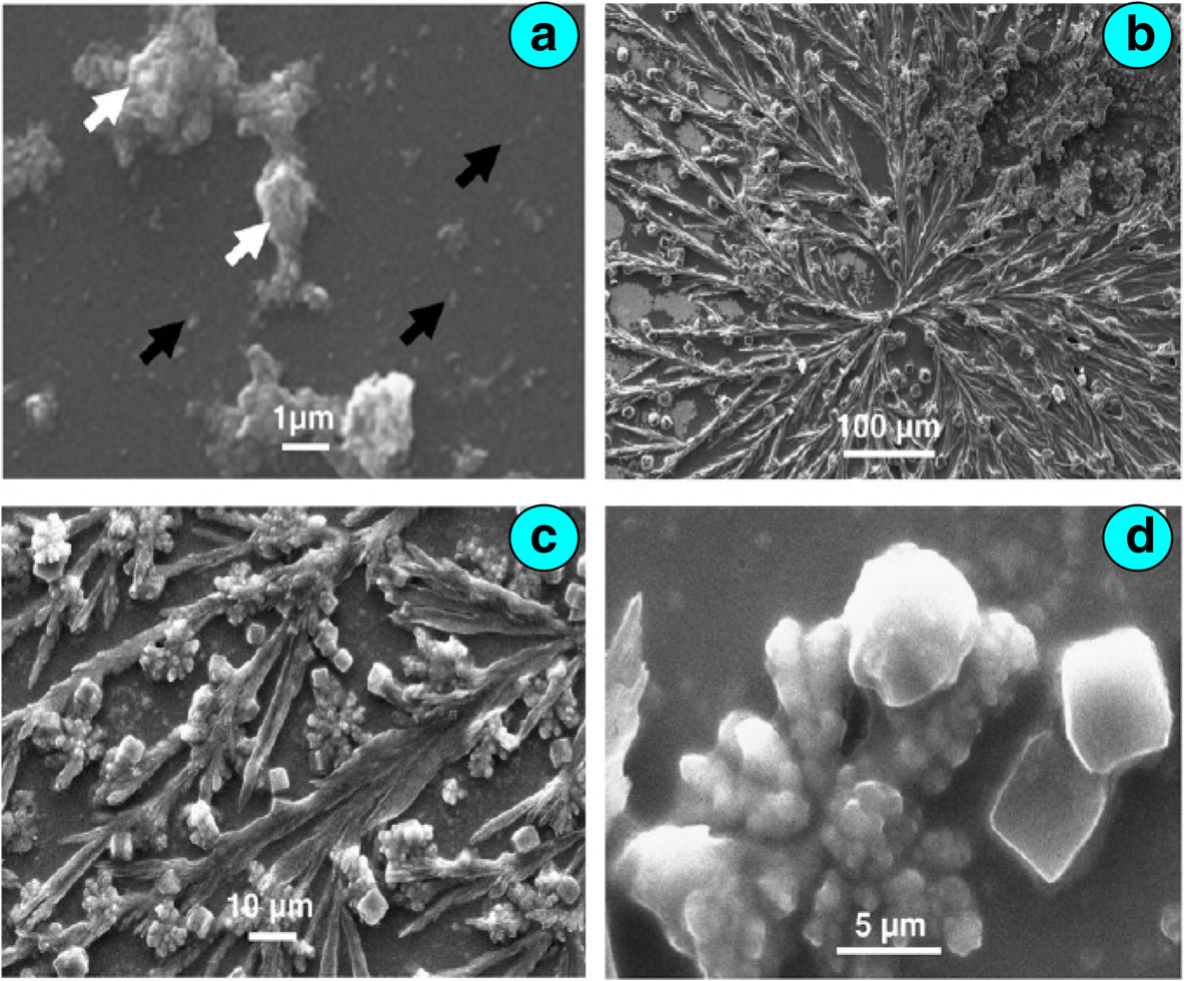
Scanning electron micrographs of **a** palladium nanoparticles. **b–** **d** Microwire networks at the periphery due to coffee ring effect. **a** Magnification: ×10,000, *inset bar*: 1 μm. **b** Magnification: ×200, *inset bar*: 100 μm. **c** Magnification: ×1000, *inset bar*: 10 μm. **d** Magnification: ×4500, *inset bar*: 5 μm [33]

Biogenic synthesis of palladium nanoparticles has been done from degradable banana peel extract and characterized them via UV-vis, IR, SEM and XRD [33]. The peel extract powder reacted with PdCl_2_ at 80 °C for 3 min in water. The UV-vis spectra of all mixtures showed a peak at 400 nm, but after the reduction of Pd^2+^ to Pd^0^, the peaks were either shifted or disappeared with a constant change in colour from yellow to red due to excitation of surface plasmon vibration in the palladium nanoparticle. The SEM images showed nanoparticles and aggregates. After accumulation, the dendrites are formed which look like a beautiful flower twig. However, at higher magnification, dendrites are shown to be composed of microcubes, nicely arranged as a motif (Fig. 5a–d). The average size of palladium nanoparticle was 50 nm. The FTIR spectral data showed the presence of carboxyl, amino and hydroxyl groups which are supposed to be active ingredients for the reduction of PdCl_2_.

Petla et al. [34] have reported the synthesis of palladium nanoparticles from soybean leaf (*G. max*) extract. Although, the reduction started after 5 min, the characteristic absorption peak at 420 nm for Pd^2+^ disappeared completely after 48 h indicating complete conversion of Pd^2+^ → Pd^0^. The TEM micrograph showed the formation of uniform spherical particles of ~15 nm. The authors claim that only 8 out of 20 essential amino acids are IR active and they reduce the Pd^2+^ ions. They have misunderstood the fundamental basis of IR spectroscopy that any molecule which can exhibit a change in dipole moment can be IR active. It is therefore suggested that all amino acids and proteins are IR active and some of them may act as reducing agents.

**Fig. 6:**
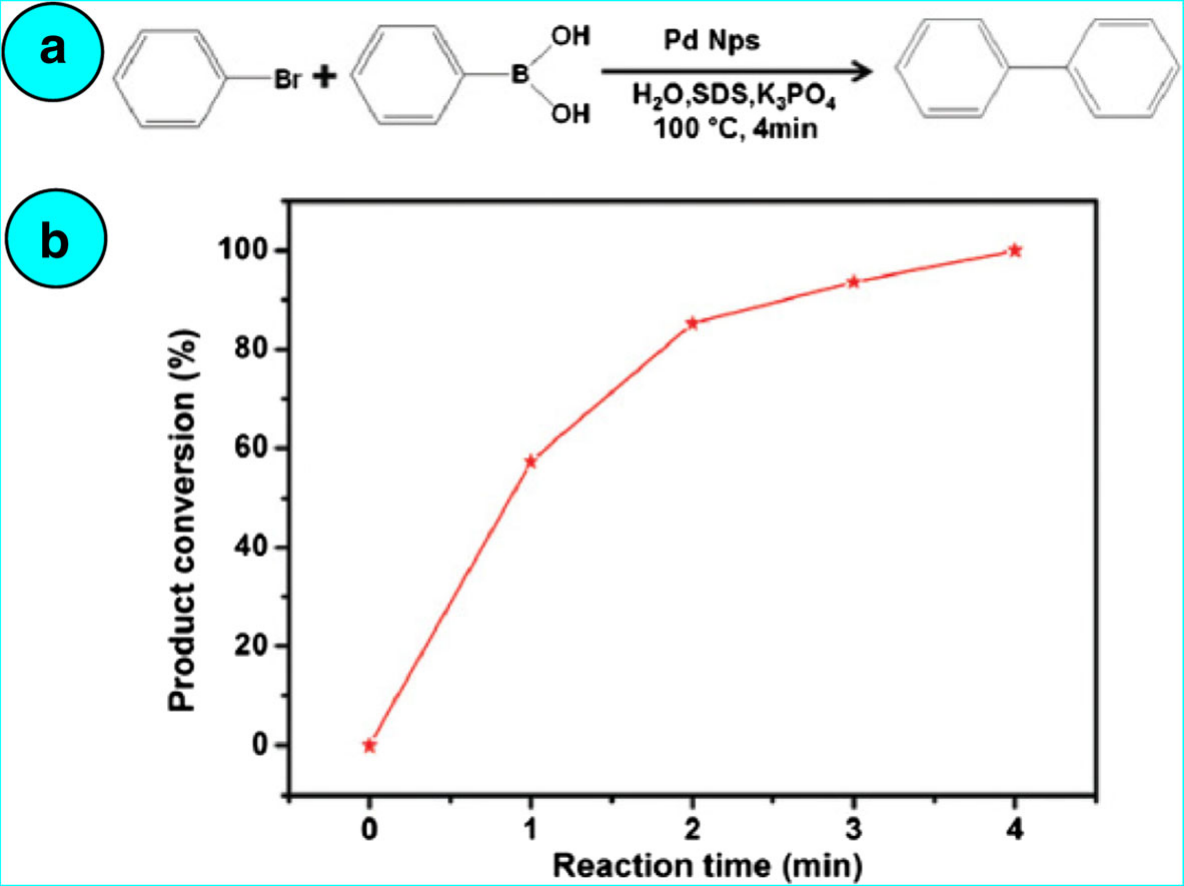
**a** Schematic representation of the Suzuki reaction of bromobenzene with phenylboronic acid under aqueous conditions. **b** Time-dependent conversion efficiency of the Suzuki reaction of bromobenzene with phenylboronic acid under aqueous and aerobic conditions determined by GC analysis [35]

Biosynthesis of palladium nanoparticles from *P. glutinosa* plant extract has been done at 90 °C after stirring the mixture of PdCl_2_+ extract for 2 h [35]. A change in colour from light yellow to dark brown showed the formation of palladium nanoparticles which was confirmed by UV-vis spectral study. TEM micrograph showed palladium nanoparticles of 20–25-nm diameters covered with organic layer from extract which act as the capping agent as well as reducing agent. The IR spectrum of the plant indicated the presence of flavonoids and polyphenols. Their catalytic activity was examined in Suzuki reaction of bromobenzene with phenylboronic acid (Fig. 6) in an aqueous medium [36] without prior activation [37] in the presence of SDS and K_3_PO_4_ under anaerobic conditions. Biphenyl was obtained when only 5 mol % of palladium nanoparticles were used as catalyst. Nearly 60 % conversion was achieved within first 1 min and was completed only in 4 min; the reaction was fast and effective.

Recently, palladium nanoparticles from *Arabidopsis* plant culture and K_2_PdCl_4_ were prepared [38]. The reduction was complete in 24 h. TEM images of different sections of the plant showed well-dispersed spherical metallic nanoparticles of an average diameter of 3 nm during first 3 h. As the incubation time increased, the size and concentration of nanoparticles also increased up to 32 nm. They were distributed uniformly in the apoplast regions. Plant had attained maximum palladium concentration after 18 h. The mechanism underlying the reduction of Pd^2+^ ion to elemental Pd inside the plant system is not yet clear. However, the binding of Pd^2+^ ions to carboxyl, amino and sulfhydryl groups, present in the plant, prior to the formation of nanoparticles is one of the likely steps. The authors have not found any enzyme in plants, and therefore, it is suggested that reduction of metal is a chemical rather than biological process. A chemical-based reduction process was carried out using a single chemical in an isolated system whereas biological reductions occur in the presence of biomolecules in a biological system such as plants or microbes. The conversion is a redox process irrespective of the chemical or biological system used.

Besides biosynthesis of palladium nanoparticles, Kora and Rastogi [39] have studied its properties as antioxidant and as catalyst. A water soluble plant gum polymer, gum ghatti (*A. latifolia*), was allowed to react with PdCl_2_ at 121 °C and 103 K Pa for 30 min which showed a change in colour followed by the disappearance of absorption peak at 427 nm in UV-vis region. The nanoparticles were spherical in shape and polydispersed, and the average size ranged between 4.8 ± 1.6 nm. Hydroxyl and carboxyl groups of the gum are supposed to be bonded to Pd^2+^ ions in the beginning which subsequently reduce and also stabilize them. The nanoparticles are believed to be stabilized and capped by proteins and polysaccharides of the gum. The present protocol of palladium nanoparticles synthesis is superior to other similar methods [33, 40] because it takes little time and produces nanoparticles of very small size (4.8 nm). Homogeneous catalytic activity of palladium nanoparticles was investigated by the reduction of dyes, for instant coomassie brilliant blue G-250, methylene blue, methyl orange and 4-nitrophenol with NaBH_4_. The characteristic absorption peaks for coomassie brilliant blue at 588 nm was monitored during palladium nanoparticles catalysed NaBH_4_ reduction. The dye decolorised within 2 min with the disappearance of the above peak showing its complete reduction in such a short span of time. Reduction of methylene blue has also been studied in the same way. Its characteristic absorption at 664 and 612 nm disappeared, and the dye became colourless showing its reduction to colourless leuco methylene blue. Similarly, methyl orange peak at 462 nm also vanished during reduction process. The reduction of 4-nitrophenol to 4-aminophenol was also monitored by examining the absorption at 318 nm which was shifted red to 400 nm due to the formation of nitrophenolate ions in the presence of NaBH_4_. With the addition of palladium nanoparticles, the intensity of the peak at 400 nm diminished with concurrent emergence of a new absorption peak at 294 nm indicating the reduction of nitrophenol to aminophenol. The conversion was also visible by the disappearance of yellow colour. The reduction of all above dyes is thermodynamically favoured but kinetically hindered due to large potential difference between donor and acceptor molecules. However, in the presence of palladium nanoparticles, both these substances are adsorbed on its surface and nanoparticles facilitate the transfer of electrons from the reductant NaBH_4_ to substrate oxidant. Since palladium nanoparticles act as redox catalyst, they decrease the activation energy of the ensuing reaction via electron relay effect [41].

Synthesis of palladium nanoparticles from *Moringa oleifera biomass containing* bis-phthalate as a natural reducing and capping agent has been reported. Their average size ranged between 10 and 50 nm. They were spherical, well dispersed and did not show any aggregation [42]. TEM studies showed a smaller size of palladium nanoparticles stabilized by the phytochemicals. It was also confirmed by the Zeta potential and GC-MS. *M. oleifera* peel extract has also been used for palladium nanoparticle fabrication [43]. They were characterized by UV-vis spectroscopy, XRD, SEM and HR-TEM studies.

Palladium nanoparticles synthesized from *Euphorbia granulate* leaf extract have been used as a heterogeneous catalyst for the phosphine-free Suzuki–Miyaura coupling reaction at room temperature [44]. TEM micrograph showed that palladium nanoparticles were 25–35 nm in size.

**Fig. 7:**
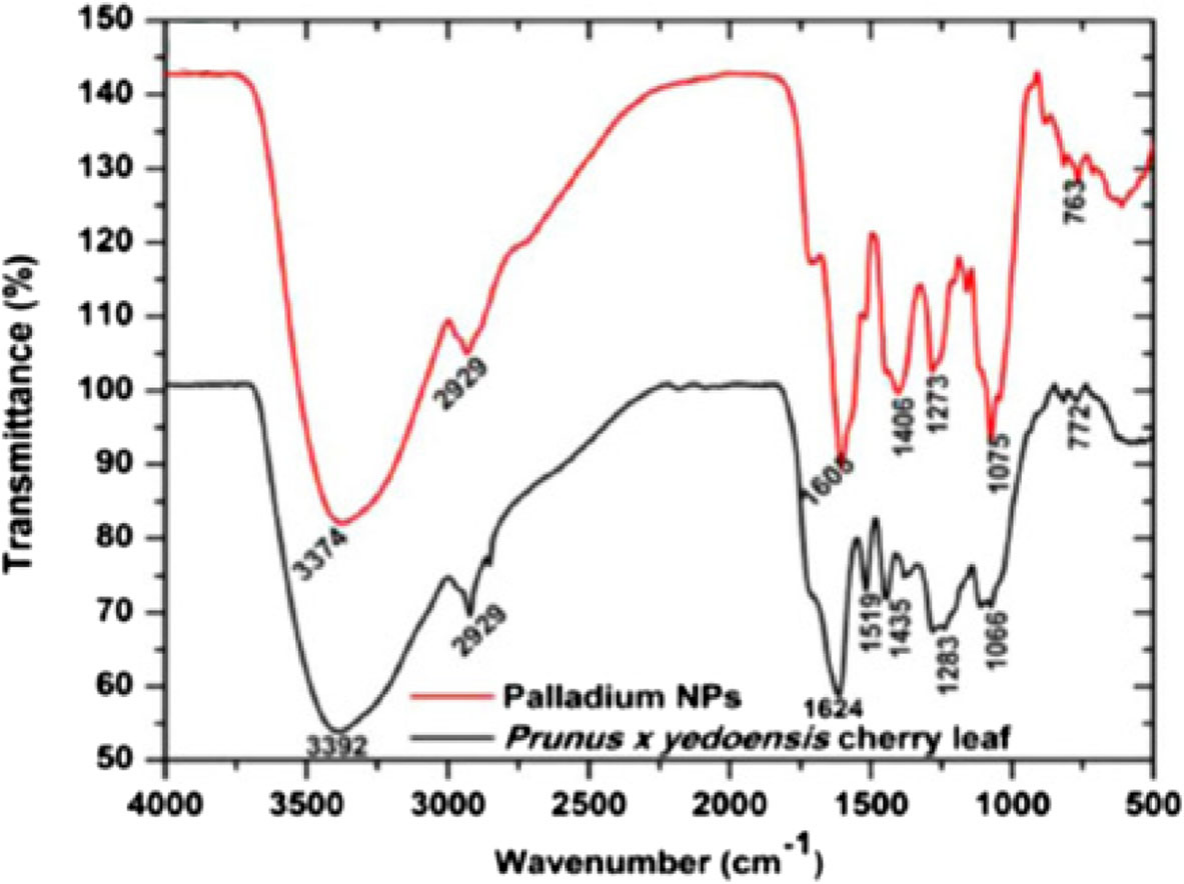
Characterization of palladium nanoparticles using FTIR studies [45]

Biosynthesis of palladium nanoparticles has been done from *Prunus* x *yedoensis* leaf extract and characterized by UV-vis, XRD, FTIR, HR-TEM and SAED [45]. Formation of palladium nanoparticles was confirmed from a change in colour from light yellow to dark brown. Manikandan et al. [45] have suggested the optimization parameters for the production of palladium nanoparticles, i.e. pH 7, 40:5 Pd(II): leaf extract, 3 mM Pd(II) and 30-min time. The UV-vis spectrum showed an absorption peak at 421 nm, XRD peak (2θ = 42.5°). XRD pattern confirmed the crystalline nature of the palladium nanoparticles. TEM images showed the particle size (50–150 nm) and their spherical shape. The FTIR spectrum of *Prunus* x *yedoensis* leaf extract (Fig. 7) showed the presence of alcohol, ethers, esters, carboxylic acids and amino acids [15, 46–48] which acted as the reducing agent to convert palladium ion to palladium nanoparticles.

### Application of Palladium Nanoparticles

Palladium adsorbs about 1000 times its own volume of hydrogen when heated to dull redness. Their catalytic activity is due to the dissociation of molecular hydrogen into atomic state: H_2_ → 2H.

Palladium nanoparticles doped with chitosan–graphene have been employed as biosensor for glucose estimation [36]. Palladium nanoparticles on graphene oxide have also been used as recyclable heterogeneous catalyst for the reduction of nitroarenes using sodium borohydride. Since the recovered catalyst can be used for five cycles, it can be used on a large-scale reduction of nitroarenes. It has also been used in the reduction of methylene blue, methyl orange and nitrophenol. The nanoparticles exhibited excellent degradation of the above dyes, and therefore, they can be used to treat the affluents containing dyes. Both palladium and platinum are extensively used in oxidative addition and reductive elimination of hydrogen. Platinised asbestos is used in many catalytic [49] reactions. For instance, (i) in the contact process for the manufacture of H_2_SO_4_, (ii) in Ostwald process for the oxidation of NH_3_ to NO for the manufacture of HNO_3_, (iii) oxidation of methanol to formaldehyde and (iv) decomposition of hydrazine to nitrogen and ammonia. Platinum-gold dendrimer-like nanoparticles supported on polydopamine graphene oxide reduce nitrophenol to aminophenol [50]. The ability to catalyse the reduction depends on platinum to gold ratios.

Palladium nanoparticles have been fabricated from *S. persica* root extract, and their catalytic activity was examined in the Suzuki coupling reactions of aryl halides with benzeneboronic acid in water to biphenyl [22]. The efficiency of the conversion rate as a function of time and yield follows the order iodobenzene > bromobenzene > chlorobenzene, although the major conversion occurred in the first 2 min. The palladium nanoparticles as catalyst can be successfully reused for only three cycles. In another study, *Myrtus communis* leaf extract was used for the production of Pd/TiO_2_ nanoparticles [﻿^51^]. Authors have demonstrated that Pd/TiO_2_ nanoparticles as a highly efficient, stable and recyclable catalyst for the ligand-free Suzuki–Miyaura coupling reaction.

Biosynthesis of palladium nanoparticles from dried fruit extract of *G. jasminoides* Ellis has been investigated for its catalytic activity by the hydrogenation of p-nitrotoluene to p-toluidine and subsequently to p-methyl-cyclohexylamine [27]. It is interesting to note that conversion of p-toluidine was 100 %, but second reduction was only 26 % at 80–90 °C. The palladium nanoparticles had been recycled five times without their agglomeration.

Kora and Rastogi [39] have used *A. latifolia* for the biosynthesis of palladium nanoparticles and demonstrated their antioxidant and catalyst potential. In many studies, palladium nanoparticles were used as catalyst for Suzuki–Miyaura reactions to synthesize pharmaceutical intermediates and other important chemicals. Palladium nanoparticles containing plant material was heated to 300 °C which contained 18 % Pd^2+^ or PdO but no palladium nanoparticles. The material Pd-300 was used as a catalyst for Suzuki–Miyaura reactions. High yield of aryliodides and arylbromides [52] were obtained which were higher than palladium nanoparticles used for similar reactions. This catalyst is far superior to commercially available palladium catalyst (10 % Pd/C) and Pd(OAc)_2_ and, hence, can be used as a potential catalyst of future.

Sheny et al. [52] have reported the biosynthesis of palladium nanoparticles from dried leaf powder of *Anacardium occidentale* at pH 6–9. TEM images showed irregular rod-shaped particles which were crystalline. They have observed that the quantity of leaf powder plays a vital role in determining the size of palladium nanoparticles. FTIR spectrum of the suspension suggested the presence of secondary metabolites having hydroxyl group which reduced Pt(IV) ions to palladium nanoparticles. These palladium nanoparticles exhibited catalytic activity in the reduction of aromatic nitrocompounds.

### Biosynthesis of Platinum Nanoparticles

Platinum nanoparticles from tea polyphenol acting both as reducing and capping agent have been fabricated [52]. These functionalized nanoparticles of 30–60 nm were crystalline in nature with face-centred cubic structure. TEM images showed that the capped nanoparticles were flower shaped. Tea polyphenols are known to contain a number of phenolic compounds which can form complexes with metal ions and subsequently reduce them to nanoparticles of different shapes and sizes [5, 53, 54].

Biosynthesis of platinum nanoparticle pellets using *O. sanctum* leaf broth was achieved at 100 °C in 1 h [55]. The reduction was quantitative and identified by a change in colour from yellow to brown and finally black indicating reduction in successive steps as shown below:

$$ \begin{array}{cc}\hfill {\mathrm{Pt}}^{4+}\overset{2\mathrm{e}}{\to}\hfill & \hfill {\mathrm{Pt}}^{2+}\overset{2\mathrm{e}}{\to}\mathrm{P}\mathrm{t}{}^{\circ}\hfill \end{array} $$

**Fig. 8:**
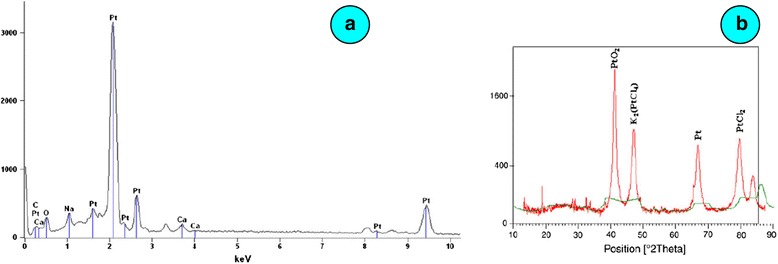
**a** EDAX spectrum. **b** XRD analysis of the reduced platinum from *Ocimum sanctum* leaf broth [55]

Ascorbic acid and terpenoids are known to be present in the *O. sanctum* leaf extract which act as reducing as well as stabilizing agents. The average particle size was found to be 23 nm. The energy dispersive absorption X-ray spectroscopy (EDAX) showed net 71 % platinum while XRD indicated the presence of PtO_2_, K_2_(PtCl_4_), Pt and PtCl_2_ (Fig. 8a, b).

A facile route for the synthesis of Pt–Au alloy nanoparticles supported on polydopamine-functionalized graphene has been reported by Ye et al. [50]. Their catalytic activity against 4-nitrophenol reduction has also been studied. Platinum exhibited higher catalytic activity than those of platinum nanoparticles deposited on reduced graphene sheets (RGO). Ascorbic acid has been used as a reducing agent instead of any natural source for nanoparticle fabrication, and therefore, this method cannot be termed “green”. It was shown earlier that the multifunctional polymer disperses the reduced graphene oxide in aqueous solution and the functional groups in the biopolymer are then bonded to metal ions and metal nanoparticles. Ye and co-workers [50] had suggested that in the case of reduced graphene oxide, coated with polydopamine, PDA/RGO containing amine and catechol groups act as a reducing agent for PdCl_4_^2−^/HAuCl_4_, followed by the reduction of ascorbic acid and production of Pt-Au-PDA/RGO. It is not a convincing hypothesis. When the functional groups on the biopolymer act as the reducing agent, obviously there is no need of ascorbic acid as a secondary reductant for the production of nanoparticles from H_2_PdCl_4_/HAuCl_4_. Besides, how ascorbic acid is reduced when it is a well-known reducing agent. Naturally, the PDA would act as a stabilizer and ascorbic acid as a reducing agent. The catalytic activity of monometallic Pt-PDA/RGO or Au nanoparticle is two to four times lower than those of bimetallic nanoparticles. During the reduction of 4-nitrophenol by NaBH_4_, the electron transfer from BH_4_^−^ to 4-nitrophenol occurred when both are adsorbed on the surface of the catalyst. Interestingly, it has been noted that the 4-nitrophenol is preferentially adsorbed on Au [56, 57].

One-pot synthesis of platinum and palladium nanoparticles has been reported from natural lignin and fulvic acid in water at pH 7 at 80 °C under aerobic conditions [58]. These polymers act both as reducing and stabilizing agents. The formation of platinum nanoparticles with lignin was followed by UV-vis spectra which showed the disappearance of a characteristic peak for Pt^4+^ at 257 nm after 4 h with a consequent change in colour from orange to dark brown. The formation of platinum nanoparticles with fulvic acid showed a band at 280 nm due to the presence of phenolic group in it. The NMR spectra showed the presence of PtCl_6_^2−^ and PtCl_5_(H_2_O)^−^ species which slowly disappear as a result of the formation of nanoparticles. TEM images showed platinum nanoparticles of irregular size which form clusters. Their average size ranged between 6 and 8 nm in diameter. Palladium nanoparticles formed with lignin and fulvic acids were always spherical and larger than platinum nanoparticles. They were of 16- to 20-nm diameter. Both platinum and palladium nanoparticles were investigated for their catalytic efficiency for the reduction of 4-nitrophenol to 4-aminophenol in the presence of NaBH_4_. The absorption peak of nitrophenol at 399 nm was diminished, and a new absorption band corresponding to 4-aminophenol appeared at 292 nm after 15 min.

Platinum nanoparticles have been prepared from polyols in the presence of poly vinyl pyrrolidone which stabilized the nanoparticles and prevented their aggregation. They were of 5–7- and 8–12-nm diameter with cubic, hexagonal, square and tetrahedral shapes [59]. Time and temperature are controlling factors for the nanoparticle formation. AgNO_3_ was added to the mixture of H_2_PtCl_6_ and polyols to control the size and shape of platinum nanoparticles.

Extracellular synthesis of platinum nanoparticles of 2–12 nm from leaf extract of *D. kaki* has been reported at 95 °C using H_2_PtCl_6_·6H_2_O as precursor [60]. Formation of nanoparticles was confirmed by a change in colour which had an absorption at 477 nm. It was also noted that 95 °C was the optimum temperature for the reduction of Pt^4+^ to Pt nanoparticles [61]. Also, the size of nanoparticles decreased with increasing temperature, perhaps due to the increased rate of reduction. The reduction is believed to be done by terpenoids and reducing sugars present in the leaf extract.

Thirumurugan et al. [62] have reported the biosynthesis of platinum nanoparticles from *Azadirachta indica* extract. TEM studies indicated the formation of polydispersed nanoparticles of small to large spheres (5–50 nm). The rate of platinum nanoparticle fabrication was increased with the increase in the reaction temperature. FTIR spectrum showed sharp peaks at 1728.22, 1365.60 and 1219.01 cm^−1^ corresponding to the presence of carbonyls, alkanes and aliphatic amines, respectively. *A. indica* leaf broth was believed to contain the terpenoids which act as the reducing agent as well as stabilizer for the nanoparticles [60].

### Application of Platinum Nanoparticles

Platinum-based nanomaterials have been shown as excellent therapeutic agents [63–70]. Platinum compounds such as cis-platin, carboplatin and oxaliplatin are frequently used in chemotherapy especially in the treatment of ovarian and testicular tumours [71].

Since platinum group compounds are cytotoxic, the tea capped platinum nanoparticles were investigated for their toxic behaviour towards human cancer cells. It was also important to examine if these are toxic to both the healthy and cancer cells similar to the platinum complexes such as cis-platin and carboplatin used in the treatment of cancer. They have many side effects like nausea, vomiting, nephrotoxicity, neurotoxicity, ototoxicity, hematuria and aloepecia. Cervical cancer cells (SiHa) were therefore treated with different concentrations of tea capped platinum nanoparticles. The influence on cell viability, nuclear morphology and cell cycle distribution showed that the proliferation of SiHa cells was inhibited by platinum nanoparticles. The tea polyphenol capped platinum nanoparticles exhibited excellent viability at concentration between 12.5 and 200 μgml^−1^ for 24 and 48 h. A significant dose dependent decrease in cell viability was noticed with increasing concentration of nanoparticles. When the concentration is enhanced, the surface area is also enhanced along with the large size of the tea polyphenol. The particle size and their agglomeration are equally responsible for the cytotoxicity of platinum nanoparticles [72].

Effect of tea polyphenol capped nanoparticles on nuclear morphology and their fragmentation has also been investigated to understand the mode of apoptosis. The fluorescence microscopic image of nanoparticles in treated and placebo SiHa cells showed deformation and fragmentation of chromatin during 24 and 48 h. However, Jensen et al. [73] and Smitha et al. [74] have shown that cell death induced by nanoparticles is solely dependent on their size, shape and surface area. Tea catechin compounds exhibit cytostatic properties in tumour cells [75, 76] and induce apoptosis in U937 cells and in human colon cancer (HCT116) cells [77]. Catechin hydrate exhibits anticancer effects by blocking the proliferation of MCF7 cells and inducing apoptosis [78].

Although platinum alloys have been used in the coronary artery disease, neuromodulation devices and catheters, [79] they are not selective for cancer because they influence both the normal cells and cancer cells, leading to many complications. Functionalized platinum nanoparticles have shown size- and shape-dependent specific and selective therapeutic properties [64, 67, 80]. In many cases, platinum nanoparticles containing other organic substances have also been used as pro-drug [67, 70, 81]. Manikandan et al. [82] have shown that small platinum nanoparticles (5–6 nm) are biocompatible and exhibit apoptosis-inducing properties [49, 83]. This ability is enhanced manifold when they are coated with polymers or fortified with phytochemicals. For instance, the herbal extracts, generally used for green synthesis of nanoparticles, contain phenols, sugars and acids which act as reducing as well as stabilizing agents. Such phytochemicals in combination with cis-platin synergise apoptosis in breast cancer and cervical cancer [52, 72, 84]. A combination of platinum nanoparticles with ion irradiation has been found to enhance the efficiency of cancer therapy [85].

## Conclusions

One-pot biogenic synthesis of palladium and platinum nanoparticles from herbal extracts, algae and fungi can be done under moderate conditions. A variety of nontoxic nanoparticles with different shape and structural motifs (spheres, rods and rings) can be fabricated and stabilized. Further, their optimization may be done by controlling the pH, temperature, incubation time and concentrations of plant extract and those of the metal salts. Application of these biogenic nanoparticles as nanocatalyst can be done in environmental remediation to scavenge the dye from the textile industries and also in the Suzuki coupling reactions for the production of many organic compounds. Fabricated nanoparticles have also shown antibacterial activity against Gram-negative and Gram-positive bacteria. The platinum group metal complexes are used as anticancer drugs, but they leave toxic effects on normal cells. It is interesting that biogenically synthesized palladium and platinum nanoparticles capped and stabilized by phytochemicals are nontoxic. The functionalized nanoparticles can be used as medicine in the treatment of cancer and also as drug carrier. A new protocol may be developed for cancer therapy using palladium and platinum nanoparticles which may be more effective and less toxic than the existing conventional drugs. Their efficacy may be increased by coating them with nontoxic and soluble biopolymers. It is sincerely anticipated that improved version of the platinum group metal nanoparticles will one day replace the conventional drugs for cancer and, also, new nanocatalyst will revolutionize the manufacture of organic compounds.
